# Video Assisted Thoracoscopic Surgical Enucleation of a Giant Esophageal Leiomyoma Presenting with Persistent Cough

**DOI:** 10.1155/2016/7453259

**Published:** 2016-02-09

**Authors:** Parvez Mujawar, Tushar Pawar, Rahulkumar Narayan Chavan

**Affiliations:** ^1^Government Cancer Hospital, Aurangabad, India; ^2^Department of General Surgery, SBH Government Medical College, Dhule, India; ^3^Asian Institute of Oncology, Mumbai, India

## Abstract

Esophageal leiomyoma is a relatively rare tumor of esophagus but it is the most common benign neoplasm of the esophagus. Small esophageal leiomyoma can be observed but larger ones and those producing symptoms should be excised. As observed for other esophageal tumors, dysphagia is its main symptom. Traditionally, open thoracotomy and enucleation are its main treatment but in the last few years video assisted thoracoscopic surgical (VATS) enucleation is gaining recognition with proven advantages of minimally invasive surgery. Herein we present our experience with patient presenting with cough rather than dysphagia as a main symptom, who was diagnosed to be having giant esophageal leiomyoma. VATS guided enucleation was accomplished successfully. Size of lesion was 16 × 4 × 3 cm. Postoperative recovery was uneventful and patient is not having any signs of recurrence, after three years during follow-up period.

## 1. Introduction

Benign esophageal tumors are relatively rare; they constitute 1% to 10% of all esophageal neoplasms [1]. Esophageal leiomyoma is the commonest benign esophageal tumor [2] which usually affects patients between 20 and 50 years of age, with male to female ratio of 2 : 1 and propensity of forming in lower two-thirds of esophagus [1]. Giant esophageal leiomyoma is defined as tumor of more than 10 cm in diameter; its incidence has been reported in 17% of cases [3]. In the past few years with development of minimal invasive surgery, video assisted thoracoscopic surgery (VATS) is getting recognition for esophageal surgery. VATS is associated with minimal morbidity compared to open thoracotomy. Herein we report a case of a young female who presented with cough as a main symptom. Diagnosis of giant esophageal leiomyoma was made which was successively treated with VATS guided enucleation.

## 2. Case Report

**Figure 1:**
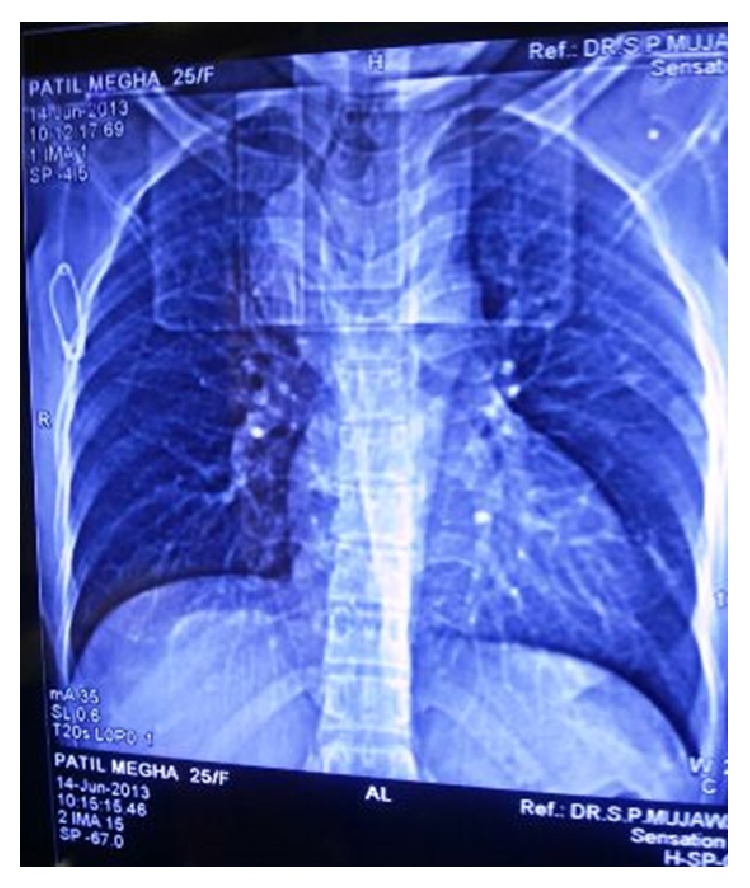
Chest X-ray showing opacity along right paratracheal region.

**Figure 2:**
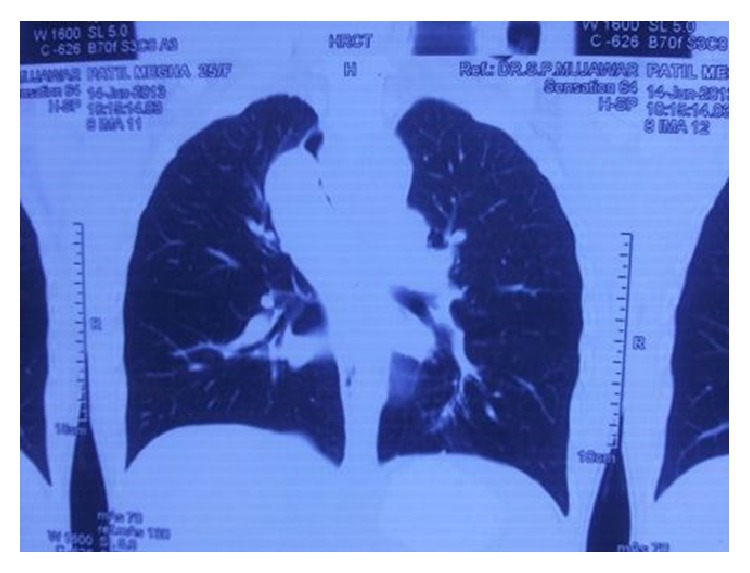
CT scan showing mass around the right side of esophagus extending to left side obliquely, suggestive of esophageal leiomyoma.

**Figure 3:**
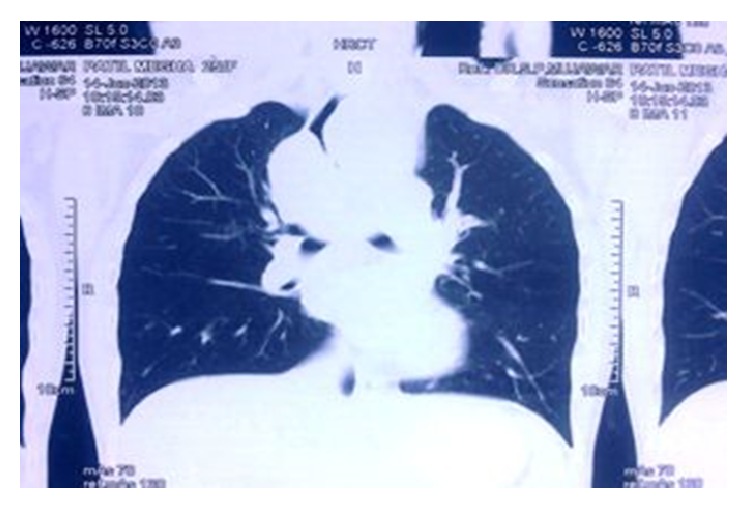
CT scan image.

**Figure 4:**
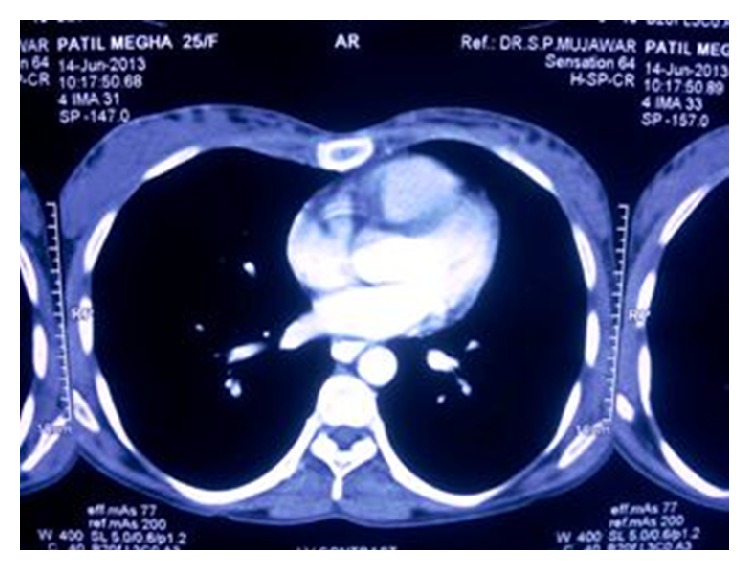
CT scan transverse section.

A 25-year-old female first presented to general physician with nonproductive cough, dull aching chest pain for 10 months with no history of weight loss, and dysphagia. Chest X-ray (Figure 1) showed opacity along right paratracheal region; correlating this feature with her symptom of cough plus high prevalence of pulmonary TB in India, physician empirically started her on AKT (anti-Koch's treatment), but her cough did not regress over next few months. Computed tomography scan (CT) of thorax revealed (Figures 2, 3, and 4) mass arising along right wall of esophagus in its upper part and showing enhancement, without paraesophageal lymphadenopathy; mass was extending up to right pleural cavity and to left side of esophagus obliquely downward, suggestive of leiomyoma of esophagus. Upper gastrointestinal endoscopy was performed which revealed a submucosal protrusion along right side of esophageal wall, without luminal compression and with intact overlying mucosa. Endoscopic ultrasound (EUS) of esophagus mentioned well-defined hypoechoic lesion arising from esophageal muscular wall suggestive of leiomyoma. Endoscopic biopsy was avoided. Routine laboratory and clinical findings were normal. Based on these findings, the decision was made to proceed with thoracoscopic enucleation after taking informed consent.

## 3. Surgical Technique 

**Figure 5:**
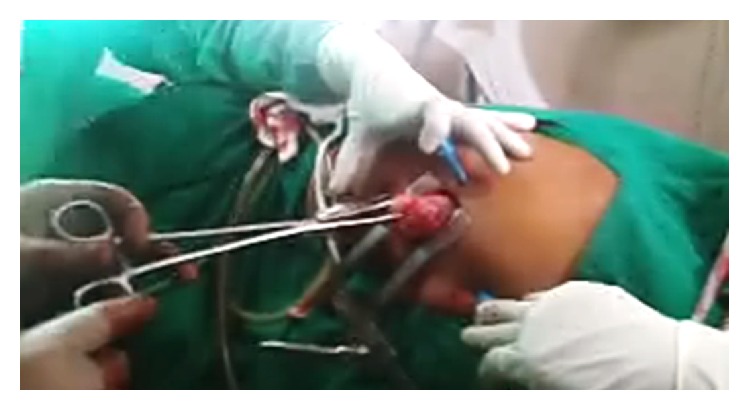
Specimen of esophageal leiomyoma being retrieved by enlarging camera port site.

**Figure 6:**
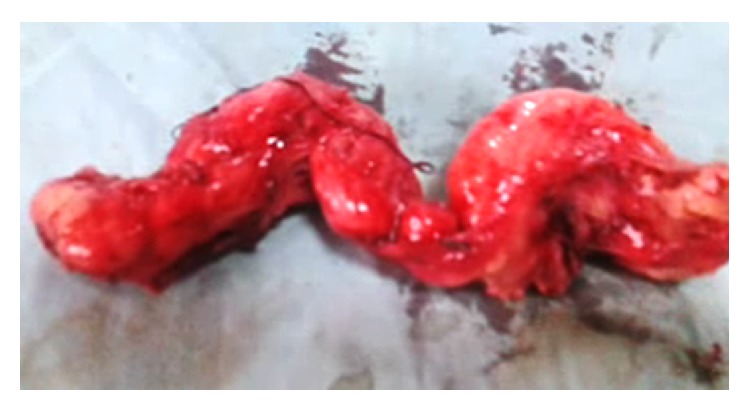
Excised specimen of esophageal leiomyoma immediately after operation.

Patient was given prone position with a pillow beneath the chest to avoid abdominal compression and to allow free diaphragmatic excursion; double lumen tube was used to collapse the right lung. Four trocars were introduced. The camera port (10 mm) was placed through a seventh intercostal space 2 cm lateral to posterior axillary line. Three 5 mm trocars were used first at the fifth intercostal space at posterior axillary line and second at ninth intercostal space one inch lateral to camera port, third port was placed approx 5 cm lateral to camera port through the same intercostal space posteriorly (Figure 3, showing postoperative wound of patient and port sites). Mediastinal pleura lateral to esophagus were incised longitudinally to expose the tumor and the adjacent esophagus. Azygos vein was identified over tumor; it was secured and cut. Esophageal myotomy about eight cm in length was performed using scissors avoiding injury to the mucosa. After blunt dissection, an avascular plane is achieved between muscle layer and the tumor, traction sutures taken to aid tumor elevation off submucosa. Dissection near hiatus minimized to prevent postoperative gastroesophageal reflux disease. After complete separation, specimen was brought out through camera port site which was enlarged up to four cm for its delivery (Figures 5 and 6). After excision of specimen, esophageal muscle layer was reapproximated meticulously using intracorporeal suture using 3-o vicryl. With the help of intraoperative endoscopy and insufflations, mucosal integrity was ensured; simultaneously any degree of esophageal narrowing following suturing of esophageal wall was ruled out. 28 No. intercostal drain was placed and complete reexpansion of lung was observed. We reinforced esophagus with parietal pleura to contain the leakage if at all it takes place.

**Figure 7:**
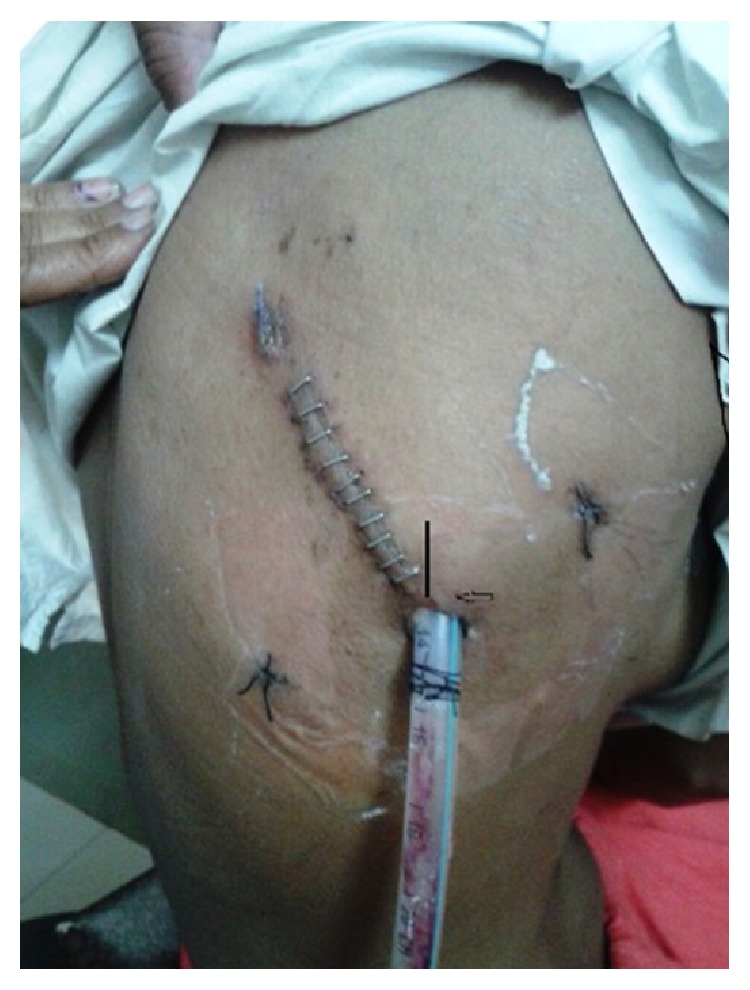
Postoperative photograph of patient showing wound and port sites. Camera port site was at lateral end of main wound.

Barium swallow at day 3 after surgery revealed no leakage and the patient was started on a liquid diet on day 4 (Figure 7). The patient was discharged on postoperative day 6. She is currently asymptomatic two years after surgery without any evidence of recurrence upon imaging studies.

Histopathological report mentioned presence of interlacing fibres of smooth muscle cells arranged in somewhat whorled appearance with areas of hyaline degeneration without any evidence of malignancy suggestive of leiomyoma of esophagus. Immunoperoxidase stainings were positive for smooth muscle actin and desmin, and negative for C-kit.

## 4. Discussion

Esophageal leiomyoma is the commonest benign tumor arising from smooth muscle cells of the esophagus. It can involve any part of esophagus but reportedly it affects distal third in 60%, middle third in 30%, and upper third of esophagus in 10% of the cases [3]. This distribution parallels the relative amount of smooth muscle cells' presence along the esophagus. It is a slow growing intramural tumor [1] which has got very limited malignant potential. Reported size of esophageal leiomyoma varies from 1 to 30 cm [1, 4]. Leiomyoma larger than 10 cm in size is called giant esophageal leiomyoma [5–8].

Often esophageal leiomyoma clinically manifests with no specific symptoms and diagnosis often is an incidental finding [9]. Though its presentation is expected to vary with size and location of tumor, still no consistent association has ever been found between these factors. Shin et al. [10] described one of the largest series of esophageal leiomyoma, with their experience clinical presentation of esophageal leiomyoma in descending order as follows: asymptomatic (58%), dysphagia (12%), epigastric discomfort (8%), dyspepsia (6%), chest discomfort (2%), and regurgitation (1%). Other rare features may be bleeding and weight loss [5–7]. Dysphagia usually appears when tumor's diameter is more than 5 cm [10]. Notably our patient presented with main feature of recalcitrant cough, and cough as a predominant or sole symptom for esophageal leiomyoma has been rarely reported. Larger esophageal leiomyoma usually grows towards outside of esophageal lumen so dysphagia need not reflect the size of tumor in such larger tumors [11].

Preoperative diagnosis of esophageal leiomyoma is often a challenge. As in our case, it can present as mediastinal mass or it may be an incidental radiologic finding. Esophagoscopy will show normal mucosa and submucosal lesion. Barium swallow is the most common imaging study advised for esophageal lesions; it will show smooth filling defect in esophageal lumen without mucosal abnormality [3]. Computed tomography (CT) and endoscopic ultrasound (EUS) are very valuable in making diagnosis, they will delineate the intramural nature of tumor without any mediastinal lymphadenopathy. Preoperative biopsy of tumor is a controversial issue [12]. In our case, we have avoided preoperative biopsy as imaging studies were diagnostic. Disadvantages reported in doing preoperative biopsy are mucosal damage while enucleating tumor and inconclusive biopsy are often due to inadequate material [11].

Consensus regarding threshold for surgical resection of these tumors has not been reached so far. As malignant change in leiomyoma is rare, some authors recommend regular follow-up for small asymptomatic tumors (<5 cm) provided malignancy is excluded [7, 12], while others suggest surgery upon diagnosis, as the possibility, even though very small, of malignant transformation is always there [11].

The conventional surgical approach especially for giant esophageal leiomyoma has been open thoracotomy or tumor resection through thoracoabdominal incision and sometimes along with gastroesophagostomy [5, 13–17]. Main morbidities of open procedures are wound pain and pulmonary atelectasis [18, 19]. Minimally invasive surgery, VATS (video assisted thoracoscopic surgery), for enucleation of esophageal leiomyoma has been reported since 1992 and it has widely gained acceptance in the last few years [18]; it avoids the morbidity of open thoracotomy. Now VATS is the preferred minimally invasive approach for enucleation of upper two-thirds leiomyoma [13, 19]. Meticulous patient selection for VATS is of utmost importance; rounded lesions where it is easy to achieve submucosal plane are ideal ones; otherwise, the option of minithoracotomy should be used. Endoscopy is very helpful intraoperatively to ensure mucosal integrity. Traction sutures make dissection easier and after enucleation muscular layer should be closed meticulously to avoid diverticular like mucosal bulging [11].

**Figure 8:**
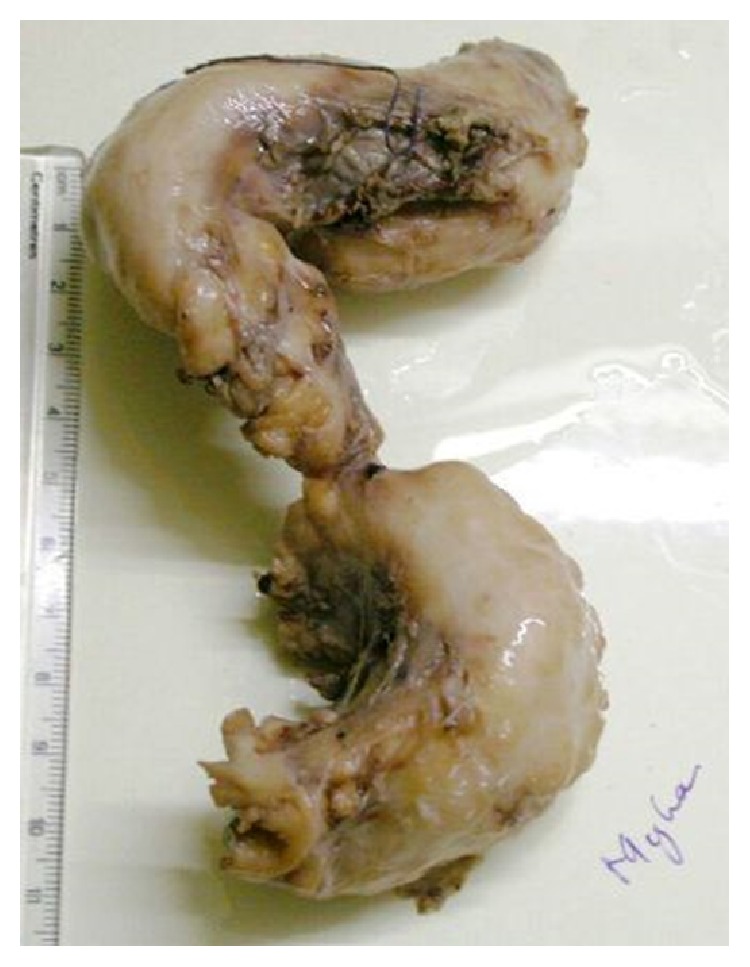
Excised specimen of esophageal leiomyoma sent for HPE after fixation, measuring approximately 16 × 4 × 3 cm.

Chen et al. [20], Kent et al. [21], and Shin et al. [10] reported their experience of thoracoscopic enucleation of giant esophageal leiomyoma of maximum size of 10 cm, 8 cm, and 4 m (mean), respectively. Notably, Hu and Lee [9] have successfully enucleated esophageal leiomyoma of maximum diameter 22 cm. In our case, maximum length of tumor immediately after excision was approximately 16 cm (Figure 8). Sun et al. [11] have recommended esophageal resection and reconstruction for giant esophageal leiomyoma instead of its enucleation, because of the technical difficulty for enucleation considering its size, possibility of malignancy, and postoperative chances of reflux esophagitis. De Giacomo et al. [1] too have described their experience of treatment of giant esophageal leiomyomas, though in their series diameter of lesion varied from 15 to 30 cm; they preferred esophageal resection rather than enucleation to approach oncologically but postoperatively no malignancy was found in any case. With our experience, VATS guided enucleation of giant esophageal leiomyoma; though challenging, it is still a safe option; it can be successfully accomplished with proper technique. And it avoids morbidity associated with major resection and open procedure. After thorough preoperative evaluation with modern imaging studies, chances of malignancy are very minimal and benefit gained with VATS enucleation, by avoiding the morbidity (due to open procedure or after partial esophagectomy), may outweigh the minimal possibility of malignancy. Still we acknowledge that a large amount of data is necessary from future number of case series to derive optimum guideline, dealing with such giant esophageal leiomyoma. With two years of follow-up for our patient she is not having any symptoms of reflux esophagitis and she is without any abnormality upon imaging studies.

## 5. Conclusion

1. VATS guided enucleation is a safe technique for giant esophageal leiomyoma; it reduces morbidity over open thoracotomy or segmental resection of esophagus.
2. Occasionally, giant esophageal leiomyoma of esophagus may mimic as respiratory tract pathology, with cough as a main symptom, so one needs to consider this possibility even in treating such patients empirically.
